# Supplementary material for: Are carbon emissions trading and green financial instruments synergistic? -Comprehensive quantitative research based on content analysis
Source: PLoS One. 2024 Mar 7;19(3):e0298601. doi: 10.1371/journal.pone.0298601 (PMC10919601; doi:10.1371/journal.pone.0298601)
Supplement: S1 Data — (ZIP) [file pone.0298601.s001.zip › Data/地级市Z_F工作报告.zip!/╔╜╢1⁄2/╚╒╒╒╩╨/└┤╫╘╣1⁄2╓┌║┼í╛┬φ┐╦╩2╛▌═°í┐_2019.docx]

各位代表：

  现在，我代表市人民政府向大会报告工作，请予审议，并请市政协各位委员提出意见。

  一、2018年工作回顾

  过去一年，面对错综复杂的国内外发展环境，全市上下坚持以习近平新时代中国特色社会主义思想和党的十九大精神为指导，全面贯彻落实习近平总书记视察山东重要讲话、重要指示批示精神，坚定不移贯彻省委、省政府决策部署，在中共日照市委的坚强领导下，紧紧围绕“一、三、五”总体发展战略，聚焦落地落实，聚力真抓实干，新旧动能转换全面起势，高质量发展扎实推进，经济社会持续健康发展。

  ——率先发展呈现新气象。预计实现地区生产总值2200亿元，比上年增长7.5%，提前两年实现地区生产总值比2010年翻一番。一般公共预算收入160亿元，在全省排名上升1个位次；增长13%，增幅居全省第1位，税收占比居全省第2位。规模以上工业增加值增长8.6%，增幅居全省前列，先进钢铁制造业成为全市首个千亿级产业集群。日照“加速度”和日照“好质量”的发展态势，增强了全市上下发力超越的信心和力量。

  ——基础设施跨上新台阶。青日连铁路正式通车，鲁南高铁将于年底通车，日照跨入“高铁时代”。日照西综合客运站建成运营，高铁、长途客运、城乡公交、旅游集散实现无缝换乘。潍日高速、204国道绕城段、迎宾路快捷路、机场快速路等重点工程竣工通车。完成港口货物吞吐量4.38亿吨，日照港跃居全国沿海港口第7位。日照机场旅客吞吐量达到90万人次，提前7年达到设计能力。日照已初步形成我国沿海重要的海陆空立体交通枢纽，为今后发展插上了腾飞的翅膀。

  ——平台打造实现新突破。日照综合保税区获国务院批复。日照高新区即将获批为国家高新区。我市成功入选国家海洋经济发展示范区。日照先进钢铁制造基地、汽车整车及零部件产业基地纳入全省重大生产力布局。日照（石臼湾）中央活力区建设迅速起势，科技馆、万达广场主体竣工，国际财富中心、日照港国贸中心等4个税收亿元楼脱颖而出。白鹭湾科技金融小镇等创新创业新载体快速发力，首个省级人力资源服务产业园建成投运，跨境电商产业园开园运营。山东外国语职业学院成功升本。日渐增多的各类平台，使我市高质量发展的承载力、集聚力不断提升。

  ——民生福祉得到新改善。民生支出突破200亿元，占一般公共预算支出80.7%，日照跻身中国地级市全面小康指数百强、民生发展百强。预计全体居民人均可支配收入25300元、增长9%，农村居民人均可支配收入提前两年实现比2010年翻一番。低保线和扶贫线“两线合一”，脱贫攻坚任务基本完成。台风抢险救灾夺取全面胜利。义务教育“全面改薄”任务圆满完成。紧密型医共体建设全面推开。群众安全感满意度居全省前列。覆盖“人的一生”民生保障网越织越密，老百姓的生活一天比一天好起来。

  一年来，主要做了以下工作：

  （一）全力推进新旧动能转换重大工程。编制实施了《新旧动能转换重大工程实施规划》，确立了“一带一路”港口枢纽、日照（石臼湾）中央活力区、先进钢铁制造基地、汽车整车及零部件基地等“十大专项”。山太飞机、国丰远大装配式建筑等15个项目，列入全省首批优选项目。装备制造业投资增长59.8%，规模以上工业利润、利税预计均增长30%左右。山钢一期一步全面投产，日钢4号ESP生产线建成投产，新落户钢铁配套企业41家。中兴汽车整车迁建项目一期基本建成，长城（威奕）汽车整车生产基地及研发中心项目签约落地，日照“整车梦”走进现实。派沃泰三工厂、威亚40万台CVT变速箱壳体加工、冠卓海工、国峤汽车座椅等项目开工建设，106项重点工业项目完成投资216亿元。全口径服务业税收突破120亿元，撑起全市国内税收收入的“半壁江山”。现代服务业投资增长34.9%，东港区获批省级现代服务业集聚示范区，日照大宗商品交易中心跻身首批省级服务业创新中心。扎实推进迎高铁时代“旅游富市”战略《行动方案》，120个旅游重点项目完成投资165亿元，东夷小镇、日照海洋公园引爆旅游新亮点，黑虎山旅游区晋升4A级景区，全市旅游消费总额达到414亿元。文化、体育、康养与旅游加速融合，幸福产业提速发展。“中国音乐小金钟”展演落户日照。成功申办第25届省运会。第24届省运会自行车皮划艇比赛、国际太极拳大赛、国民休闲水上运动会、中国体操节、国际马拉松等赛事活动圆满成功，打造了一批体育旅游精品线路、精品赛事、精品景区。

  （二）着力抓改革扩开放促创新。重点领域改革深入推进。“一次办好”改革在全省率先实现“六十证合一”，“3440”专项改革目标基本实现，企业开办“513模式”获国务院通报表彰。新增市场主体5.3万户，总数达到25.1万户。市级机构改革稳妥有序，新组建部门完成挂牌。园区职员制、薪酬制改革全面铺开。扎实开展国家标准化综合改革试点。落实降税减费政策，为企业减负29.6亿元。日钢、岚桥、五征、兴业入选中国民营企业500强。加强财政预算管理和绩效考核，莒县被国务院表彰为全国财政管理工作先进县。五莲县被评为全省首批社会信用体系建设试点示范城市。国企改革加快推进，61户权属企业完成混改。农村公共供水管理体制改革基本完成。80%以上村庄完成农村集体产权制度改革。农村产权交易中心实现“县乡全覆盖”。

  双向开放不断扩大。坚持以项目论能力、以项目论水平、以项目论作风、以项目论英雄，拼格局、拼平台、拼创新、拼环境，项目招引取得丰硕成果。全市新招引过亿元项目231个，其中世界500强企业7家、中国500强企业9家。我市入围中国外贸百强市。成功举办青年企业家创新发展峰会日照行、儒商大会日照发展恳谈会、日照国际友好合作交流周等活动，向海内外展示了日照对外开放新气象。港口带动持续发力，我市被列为港口型国家物流枢纽承载城市。 “东煤南移”、铁路货运“南进南出”稳步推进，日照港新开至日本九州等集装箱航线9条、至莫斯科等集装箱班列6条、无水港5个。经济合作不断深化，与中冶集团、保利集团、中国重汽、山东广电、齐鲁交通发展集团、省土地发展集团、兖矿集团等签署战略合作协议，与临沂市、吕梁市达成战略合作，与瓦日铁路沿线14座城市结成旅游、报业等战略联盟，与法国旺多姆市缔结友好城市，日照的“朋友圈”越来越大。

  创建国家创新型城市全面启动。制定了创建实施方案，全市兴起了创新发展热潮。全社会研发投入占GDP比重提高幅度居全省第1位。高新技术企业发展到144家、增长56.5%，增幅居全省第2位。五征、金禾博源跻身全省高新技术企业创新能力百强；创泽智能机器人被认定为全省首批“瞪羚示范企业”。国家碳素结构钢质检中心建成，上海交大海洋智能装备演进中心、钢铁研究院、山大智能制造研究院等落户日照，新增省级以上创新平台15个、院士工作站7个，引进高层次创业人才（团队）44个、创新团队57个、创新人才814名。评选首届日照工匠10人。“技能中国行”2018—走进山东活动在日照举办。日照职业技术学院入选第一批全省优质高职院校建设学校。市技师学院被认定为全省技工教育特色名校。

  （三）深入实施乡村振兴战略。坚持全域化谋划、市场化运作、项目化推进、精品化打造，乡村振兴实现良好开局。大力发展现代农业。新型农业经营主体发展到9200余家。特色产业种植面积超过150万亩，“南茶北引”获评山东省庆祝改革开放40周年最具影响力事件之一，日照绿茶入围2018中国品牌价值区域品牌百强榜。创建国家级海洋牧场4处、省级海洋牧场示范项目11处。着力打造新型载体。新建市级田园综合体5个、现代农业产业园10个、市级特色小镇6个。东港区创建为山东省休闲农业和乡村旅游示范县区。西湖镇、巨峰镇、招贤镇、松柏镇和叩官镇，列入全省乡村振兴示范乡创建名单；有50个村进入示范村创建行列。碑廓镇创建为山东特色产业镇“乡村振兴示范镇”。龙门崮入选省级田园综合体，五莲县现代农业产业园进入省级创建名单。莒县赵家石河村入选第五批“中国传统村落”。建成凤凰措、小茶山等精品民宿11个，不负艺术客栈入选全省五星级精品民宿。建成市级美丽乡村306个、省级美丽乡村示范村20个。新增农村“幸福公路”1000公里，五莲县成功创建全省首批“四好公路”示范县。完成168个农村集体经济空壳村“清零”。

  （四）坚决打好污染防治攻坚战。深入开展环保督察“回头看”，持续抓好整改任务落实。“四减四增”三年行动全面启动，“蓝天、清水、净土、绿盾”行动扎实推进，日照成为国务院通报表彰的全国4个环境质量改善成效明显的市（州）之一。新一轮林水会战造林15万亩、建设水利工程118项。“五大碧海行动”退渔还海1450亩，修复海岸线10.4公里。出台了建设扬尘、渣土车管理办法，市区主要空气污染物平均浓度持续下降，空气质量优良率稳居全省第一方阵，老百姓的蓝天幸福感不断增强。“清清河流”行动完成河道治理34项。新增污水管网114公里。省控以上重点河流断面水质全部达标。四级“河湖长”体系全面建立，山水林田湖草系统治理扎实推进，建成省级以上湿地公园9处，傅疃河口湿地公园获批国家湿地公园。东港区、五莲县跨入省级生态区县行列，国家和省级生态乡镇分别达到20个、41个。

  （五）大力提升城市承载能力。突出交通基础设施互联互通、日照西综合客运站片区环境提升、铁路沿线环境整治三项重点，集中开展“百日会战”，高标准、高效率建设天津路、秦皇岛路和站前广场等“城市新客厅”。《日照市城市总体规划（2018—2035年）》获批。82项市级重点城建工程预计完成投资80亿元。新建改造城市道路131条。国家生态园林城市创建全面启动，建设市民公园等绿化工程438处，新增绿化面积279万平方米。整治改造老旧小区67个。拆迁改造城中村54个、房屋1.6万处。开工建设棚改安置房8.6万套，开工率100%。出台城市管理综合考核办法，对全市55个街道乡镇分类进行考核排名，城市精细化管理水平明显提升。

  （六）持续强化公共服务。加大社会民生保障力度。发放创业担保贷款2.4亿元，支出就业创业资金1.2亿元。城镇新增就业4万人，登记失业率2.25%。新增各类参保人员14.7万人。企业最低工资标准提高到每人每月1730元。城乡低保分别提高到每人每月550元、395元。创新灾害救助模式，全面启动民生综合保险试点。投资26.2亿元，新建、改扩建幼儿园100所、中小学39所，新增校舍面积45万平方米，普惠性幼儿园覆盖率达到80%以上。东港区、莒县特殊教育学校启用。市人民医院、市中医医院、莒县中医医院通过三甲复审，日照心脏病医院启用。在全省率先为独生子女家庭和失独及伤残家庭，购买意外伤害及住院护理补贴保险。43处乡镇敬老院完成社会化改造。新增新能源公交车210辆，实现城乡公交全覆盖。改造农村危房3279户、农厕6.4万户。完成农村清洁取暖工程2.5万户。

  各项事业协调发展，社会治理创新不断加强。《日照市文明行为促进条例》《日照市饮用水水源地保护条例》颁布实施。新时代文明实践中心建设试点扎实有效，五莲县获批全国试点县，岚山区获批省级试点县。县级融媒体中心建设全面启动。涌现出“最美人梯”救人群体等一批身边好人和先进典型，“摆渡”爱心食堂获中国青年志愿服务金奖，“礼让斑马线”成为街头靓丽风景线，一件件细微小事，让整座城市充满温情、更有温度。荣获“文化强省”建设先进市，实现市县区满堂红。市文博中心开工建设。夏立君散文集《时间的压力》获第七届鲁迅文学奖。现代吕剧《山东汉子》获国家艺术基金支持。《日照市志》二轮修编完成。新建全民健身工程255处，群众体育工作经验在全国会议上作典型发言。贸易融资风险基本化解，重构地方金融生态破冰前行。“七五”普法通过省级中期验收，党政一体法律顾问、一村（社区）一法律顾问基本实现全覆盖。强力推进扫黑除恶专项斗争，扎实开展创建“平安海区”专项整治，圆满完成全国“两会”、上合组织青岛峰会安保维稳任务。食品药品质量监管不断加强，被评为“山东省食品安全示范城市”。生产安全事故起数、死亡人数实现“双下降”，社会大局保持和谐稳定。国防动员、双拥共建和民兵预备役建设持续加强。新建城市社区20个、农村社区17个。对口支援新疆麦盖提县、扶贫协作重庆黔江区工作扎实有效。妇女儿童、民族宗教、外事侨务、对台事务、国家安全、人民防空、应急管理、防震减灾、气象水文、慈善老龄、残疾人、红十字、档案、仲裁、打私等工作都取得了新成绩。

  （七）切实加强政府自身建设。把抓好党建作为最大政绩，完善政府党组理论学习中心组学习制度，制定政府党组落实意识形态工作责任制实施方案，推动党中央和省委、市委决策部署落地见效。自觉接受人大依法监督和政协民主监督，支持各民主党派、工商联、无党派人士参与重大决策民主协商。办复人大代表建议180件、政协提案363件，办复率、面办率、满意和基本满意率100%。建立政府系统重点项目、重点工作定期调度、定期通报制度。扎实做好政务公开工作，在全省首推市民代表列席政府会议制度，政务信息系统整合共享实现省定目标。加强政府公信力建设，守信联合激励和失信联合惩治机制全面建立。深入开展“大学习、大调研、大改进”，组织赴青烟威等地考察学习，举办《问政日照》6期，持之以恒解放思想、转变作风。坚决做好中央巡视“回头看”反馈意见整改，加强审计监督，政风持续好转，行政效能进一步提升。认真贯彻执行中央八项规定及实施细则精神、省委市委实施办法，深入推进反腐倡廉，日照港整顿改革取得重要成果，重点领域专项治理成效明显，清廉日照建设迈出坚实步伐。

  各位代表！过去一年的成绩来之不易，这是习近平新时代中国特色社会主义思想科学指引的结果，是省委、省政府正确领导和关心支持的结果，是市委团结带领全市人民砥砺前行、共同奋斗的结果，是市人大、市政协和社会各界有效监督、大力支持的结果。在此，我代表市人民政府，向全市人民，向市人大代表和政协委员，向各民主党派、工商联、无党派人士、各人民团体和社会各界人士，向驻日照人民解放军指战员、武警官兵、公安干警和消防救援队伍指战员，向国家和省属驻日照单位、省“千名干部下基层”服务队，向所有关心支持日照发展的海内外朋友，表示衷心的感谢和崇高的敬意！

  我们也清醒认识到，我市发展还面临不少困难和挑战，政府工作也存在一些短板和不足。外部环境复杂严峻，我国经济运行稳中有变、变中有忧，我市经济面临下行压力，实体经济比较困难，个别经济指标距离去年年初预期目标有差距；发展不平衡不充分的问题突出，综合实力不强，产业规模不大、结构不优并存；基础设施建设仍有不少瓶颈，区位、生态、港口、海洋等优势尚未充分释放；人才、科技、开放等存在突出短板，创新能力不强，新旧动能转换任务艰巨；部分领域风险隐患不容忽视，安全生产形势依然严峻，生态环保存在薄弱环节，有些区县、园区政府隐性债务偏高；教育、医疗、养老等民生领域，优质公共服务供给不足，一些老百姓的烦心事、操心事解决得还不够好；少数公务人员作风不实、能力不足，不想为、不敢为、不能为现象还不同程度存在，等等。问题是时代的声音。我们必须牢记职责使命，尽心尽力加以解决。

  二、2019年经济社会发展主要目标和总体要求

  今年是新中国成立70周年、日照市建市30周年，是全面建成小康社会的关键之年，也是我市加快新旧动能转换、实现高质量发展的重要一年。做好今年的政府工作，意义重大，使命光荣。政府工作的总体要求是：以习近平新时代中国特色社会主义思想为指导，认真贯彻党的十九大、十九届二中、三中全会精神，深入落实习近平总书记视察山东重要讲话、重要指示批示精神，加强党的领导，坚持稳中求进工作总基调，坚持新发展理念，坚持推动高质量发展，坚持以供给侧结构性改革为主线，坚持深化市场化改革、扩大高水平开放，坚持“创新、落实”工作主旋律，以新旧动能转换重大工程为引领，深入实施“一、三、五”总体发展战略，全力推动“城市品牌建设、特色产业集群建设、平台建设、项目建设、人才队伍建设”和“双招双引、改革开放、乡村振兴、民营经济、改善民生”十大突破，统筹推进稳增长、促改革、调结构、惠民生、防风险工作，进一步稳就业、稳金融、稳外贸、稳外资、稳投资、稳预期，加快塑造高质量发展新优势，促进经济持续健康发展和社会和谐稳定，以优异成绩庆祝中华人民共和国成立70周年、日照市建市30周年。

  各位代表，站上而立之年的新起点，日照以什么样的新姿态强起来、富起来、美起来，全市人民充满新期待。我们必须牢牢把握重要战略机遇期，深刻把握区域竞争新趋势，在更高水平上谋划日照未来，扎实推进“五城同创”，擦亮城市品牌，提升日照的知名度、影响力和竞争力。

  ——加快打造创新之城。以创建国家创新型城市为契机，推进理念、平台、产业、人才、制度“五大革新”，激活创新第一动力，用好人才第一资源，让创新成为日照最强劲的引擎。

  ——加快打造生态之城。以创建国家生态园林城市为契机，推进生产美、生活美、生态美，全力增加绿色空间，全面提升绿色品质，全民共享绿色福祉，让整座城市绿意盎然、鸟语花香。

  ——加快打造活力之城。以承办第25届省运会和争创全民运动健身模范市为契机，把发展群众体育、办好赛事与培育产业深度融合，精心打造品牌赛事，积极探索“体育+”新业态、新模式，让城市动起来、活起来、人气旺起来。

  ——加快打造旅游之城。以创建国家全域旅游示范区和中医药健康旅游示范区为契机，突出全域、精品，加快补齐短板，推动旅游全景区打造、全方位提升、全要素配套、全产业融合、全社会参与，让“阳光海岸·活力日照”品牌更加响亮。

  ——加快打造蓝色之城。以建设国家海洋经济发展示范区为契机，增强经略海洋意识，坚持科学用海，做强港口物流，壮大海洋产业，突破湾区经济，推动“港产城海”融合发展，让海洋真正成为日照高质量发展的战略要地。

  今年经济社会发展的主要预期目标是：地区生产总值增长7.5%左右，一般公共预算收入增长10%左右，固定资产投资增长7%左右，社会消费品零售总额增长8%左右；全体居民人均可支配收入增长8.6%左右；城镇登记失业率3.5%以内，居民消费价格涨幅3%左右；全面完成省下达的节能减排降碳约束性指标和环境质量改善目标。

  各位代表，展望2019年，机遇与挑战并存，动力与压力同在。从蓄势积能到发力超越，唯奋斗者进，唯实干者上，唯搏击者胜，必须旗帜鲜明地唱响“创新、落实”工作主旋律。只有创新创新再创新，才能化挑战为机遇，推动创新发展、持续发展、领先发展；只有落实落实再落实，强力推进“十大突破”，才能把握当下、成在实处，变蓝图为现实。具体工作中，将把握好以下几点：第一，始终坚持旗帜鲜明讲政治，把思想认识高度统一到中央、省委省政府和市委对当前形势的判断上来，把党的领导贯穿到政府工作的各方面、全过程，扎实办好日照的事情。第二，准确把握从“有没有”到“好不好”这个根本转变，铆定新旧动能转换主抓手，始终在高质量发展上聚焦用力，加快质量变革、效率变革、动力变革。第三，坚持问题导向、创新导向、市场导向，更加自觉地用足用好改革开放“关键一招”，敢领改革之先，勇立开放潮头，提升用市场化、法治化手段推动发展的本领。第四，更加注重打基础利长远、补短板强弱项，保持定力、久久为功，把日照的区位优势、生态优势、港口优势、海洋优势呵护好、发展好、转化好，把长远发展的基础打牢实。第五，牢牢把握“抓落实、办实事”的工作定位，勇当新时代泰山“挑山工”，让真抓实干蔚然成风，创出经得起人民、实践、历史检验的业绩。

  三、2019年政府工作重点任务

  今年，我市改革发展稳定任务艰巨而繁重。必须坚持项目化、工程化，统筹兼顾、突出重点、精准施策，扎实做好以下工作：

  （一）立足实现高质量发展，在推进新旧动能转换上加力增效。认真贯彻落实中央“巩固、增强、提升、畅通”八字方针，深化供给侧结构性改革，以更大力度推动新动能多点发力、重点突破。

  强化科技创新引领。抓好创新型区县、园区、企业建设，战略性新兴产业增加值占GDP比重达到8%，研发投入占GDP比重提高0.2个百分点，高新区跨入国家级行列。力争高新技术企业净增50家，新认定市级及以上“专精特新”企业20家，培育“瞪羚”“隐形冠军”企业15家。着力发展“四新”经济，围绕新一代信息技术、新材料、人工智能、区块链等领域，实施30项科技重大专项，启动5G通讯布网，推动一批重点企业“机器换人”“两化”融合。着力突破一批创新平台，建成中科院山东综合转化中心日照中心，支持企业建设研发平台。大力实施“日照英才”工程，完善人才服务保障体系，力争引进更多的高层次创业创新团队、创新人才。完善现代职业教育体系，推动产教融合、校企合作，加快培养紧缺技能人才。支持日照职业技术学院创建国家特色高水平高职院校、日照技师学院创建全国一流技师学院。支持驻日照高校发展，建设“城市+大学”共同体。评选新一届“日照工匠”。深入开展质量提升行动，推进国家标准化综合改革试点工作，支持企业提品质、创品牌，培育“百年老店”。

  打造世界一流海洋强港。港口是日照最核心的战略资源、最重要的发展平台、最靓丽的城市名片。对标世界一流海洋强港，全力推动诚信港口、智慧港口、高效港口、绿色港口“四型港口”建设。加大港口基础设施建设力度，加快“东煤南移”、日照钢铁精品基地配套码头、第四个30万吨级原油码头和深水集装箱码头等重点工程建设。实施石臼港区主要疏港道路畅通工程，加快岚山疏港铁路、岚罗高速等项目建设，完善港口集疏运体系。强化内外联动，拓展港口发展空间，积极对接瓦日、新菏兖日铁路沿线城市，广布无水港；开拓海外航运市场，增开新的航线和班列，支持日照港境外上市、融资扩股。强化港口和城市互动，加快实施海龙湾退港还海工程，用好港口展览馆、老灯塔、兴港纪念碑等资源，让游客进得来、能体验。

  加快中央活力区建设。重点培育总部经济、商务金融、科技创新、博览会展等8大新兴业态，加快推进日照综合客运站、海韵广场、沪港中心等项目建设，建成科技文化中心、科技馆、黄海之眼、万达广场、兴业喜来登酒店等项目，海曲路地下通道竣工通车。抓好国际财富中心、日照港国贸中心等商务楼宇招商入驻，推动“一楼一特色、一楼一园区”。力争中央活力区重点城建项目完成投资25亿元以上，税收亿元楼10座以上。

  做强特色产业集群。支持山钢增量崛起、日钢转型发展，推动“纵延横联”，提升产业链水平。年内山钢一期二步主体工程贯通，启动二期工程；日钢第五条ESP生产线建成投产。加强钢铁配套产业规划建设，加快国家级钢铁产业研究院、金属表面处理中心等公共服务平台建设，力争新引进“吃钢嚼铁”及配套项目20个以上，钢铁产业产值达到1150亿元。开工建设长城（威奕）汽车整车生产基地及研发中心项目，中兴汽车整车项目一期全面投产，支持五征、威亚等骨干企业升级。举办先进钢铁制造、汽车整车及零部件等发展论坛或研讨会。用好国家海洋经济发展示范区“金字招牌”，高标准建设国家海洋牧场，带动海洋“新六产”发展；推进海洋装备制造、海洋生物医药等产业发展，培育特色新兴产业。抓好化工产业安全生产转型升级专项行动，提升岚山和莒县海右化工园区建设水平，下决心推进“腾笼换鸟”。实施新兴产业倍增计划，推动生物医药、电子信息、军民融合等特色产业园发展。抓好百项重点工业项目，力争新增规模以上工业企业100家，规模以上工业增加值增长9%左右。

  推动幸福产业倍增发展。大力培育旅游、康养、体育、文化等新兴消费热点，做好跨界融合文章。深入实施“旅游富市”战略，办好全国精品民宿发展研讨会，打造阳光海岸带精品旅游线路。推进万平口创建5A级景区、山海天创建国家级旅游度假区。扎实推进东方太阳城、莒国古城、金帆山海汇等重点旅游项目建设，加强与大型文旅企业对接合作，力争引进一批高端文旅项目，建成太公旅游小镇等精品项目，给游客更多“非来不可的理由”。围绕“办好一次会，搞活一座城”，精心筹办、扎实备战第25届省运会，改造提升一批体育设施，推进日照奎山体育中心、网球公园等体育场馆建设，香河体育公园建成投运；办好太极拳、水运会、马拉松、体操节和围棋大会、青运会网球预赛等重大赛事。扎实推进一批康养重点项目，加快发展医养健康产业。深化“放心消费诚信日照”创建活动，营造安全放心的消费环境。

  （二）立足增强发展动力活力，在深化改革扩大开放上加力增效。坚定不移抓改革促开放，努力让改革的路越走越宽，开放的门越开越大，打造新旧动能转换、高质量发展的强劲引擎。

  着力推进市场化改革。对标世界银行营商环境评价指标体系，突出法治、诚信、公平，推动“放管服”改革迈向新高度。推进相对集中行政许可权改革，实现“一枚印章管审批”。在各级政务服务中心设立审批服务代办窗口，健全代办机制，对企业办理特定领域相关审批手续提供全程代办、无偿代办。深化“3440”专项改革，巩固提升企业开办“513”模式，全面推开“证照分离”，加强“双随机一公开”监管。深入推进“互联网+政务服务”，实现政务服务事项数据“全打通、全归集、全共享、全对接”。扎实推进市县机构改革，科学编制涉改部门“三定”规定，稳妥做好人员转隶，建立规范高效的运行机制。深化扩权强县、扩权强镇改革。鼓励企业直接融资，力争有更多的企业挂牌上市。深化国有资本投资、运营改革，积极稳妥推进国企混改，增强市属国企搏击市场、可持续发展能力。

  扩大高水平对外开放。加快构建毗邻城市、铁路沿线城市、“一带一路”国家和地区“三个圈层”，落实好已签订的区域合作协议，力争再添一批战略合作城市。加大“双招双引”力度，推动重大活动签约项目落地，引进一批好项目、大项目。把日韩作为主攻方向，依托现有韩资、日资企业，推动中韩汽车零部件产业园、中日健康食品产业园发展，力争实际使用外资有较大增长。积极应对中美经贸摩擦，精心组织我市企业参与“境外市场开拓百展计划”，创新境外参展办展方式，开展日照品牌“海外行”活动。落实优化口岸营商环境、促进跨境贸易便利化政策措施，进一步压减整体通关时间，降低通关成本。积极稳妥推进综保区建设，力争上半年完成封关验收。以创建省级跨境电商综合试验区为抓手，加快培育外贸新业态新模式，促进外贸转型升级。坚持“走出去”“引进来”相结合，支持日照港、岚桥港与境外港口互通合作，办好五征—乌干达农业合作示范区。把园区作为开放的主阵地，加快实施新一轮园区提升行动，以去行政化为方向，推广“管委会+公司”模式，创新投融资机制，增强园区发展活力。坚持“亩均论英雄”，引导园区产业集群化、差异化、高端化发展。推动岚山开发区创建国家级，力争空港经济开发区获批省级。加快推进空港口岸开放，力争年内开通国际包机航线。

  大力支持民营经济和外资企业发展。全面落实扶持民营经济发展的各项政策，出台进一步支持民营经济高质量发展的若干措施，开展“送政策上门”活动。完善民营企业服务体系，建立健全领导干部联系企业、民营经济发展联席会议制度，畅通政府与企业沟通渠道，开展政策执行第三方评价。落实减税降费政策，从破解融资难题、鼓励创新发展、培养企业家队伍等方面，给予实实在在的帮助。实施骨干企业培育工程，开展中小微企业、高成长性中小企业培育提升专项行动，深入开展“个转企”“小升规”工作，力争完成规范化公司制改制56家。建立实施信用“黑名单”、企业信用修复制度。保护民营企业家和外商合法权益。加强对民营企业、外资企业的管理服务，引导企业依法合规经营。着力构建“亲”“清”新型政商关系，制定政商交往正面清单和负面清单，为广大干部服务企业、与企业家交往划定“安全区”。

  （三）立足筑牢小康社会基础支撑，在打好“三大攻坚战”上加力增效。针对突出问题，打好重点战役，加快形成标本兼治、系统发力的良性机制。

  打好防范化解重大风险攻坚战。落实地方金融监管责任，严厉打击非法集资、金融诈骗等违法犯罪活动。坚持“一企一策、一企一专班”，推动担保圈核心企业“破圈解链”。加大逃废银行债务打击力度，优化金融生态环境。稳妥处置政府债务风险。严格落实安全生产责任制，实施安全监管“实名制”。推进安全风险管控和隐患排查治理双重预防体系全覆盖。深化重点行业领域专项治理，坚决遏制重特大安全事故发生。成立重点行业领域安全指导服务专家小组，推行第三方监管服务、城市安全风险评估，提高本质安全水平。加强全社会安全教育，健全群众有奖举报、企业内部举报等制度，打赢安全生产人民战争。

  打好精准脱贫攻坚战。坚持“两不愁、三保障”现行标准，巩固提升脱贫攻坚成果，确保政策不变、投入不降、力度不减、队伍不撤。开展建档立卡提升行动，做到精准到户、精准到人、精准到每一笔收入。强化已建扶贫项目管理，因地制宜发展特色优势扶贫产业，提升产业扶贫质量。建立脱贫监测和即时帮扶机制，对因病因灾出现的临时性、突发性贫困人口，分类施策、精准帮扶，确保全面小康路上一个不少、一个不落。开展扶贫扶志行动，激发贫困群众内生动力。实施村集体经济清零倍增工程，力争集体收入10万元以上行政村占比超过60%。加强扶贫领域腐败和作风问题专项治理，为打赢脱贫攻坚战提供坚强保证。

  打好污染防治攻坚战。践行“绿水青山就是金山银山”理念，开展生态文明建设示范区县创建活动。抓好青龙湖山水林田湖草综合发展示范区规划建设。全力推进“四减四增”三年行动计划，继续实施“蓝天、清水、净土、绿盾”等行动，打好蓝天保卫战、黑臭水体整治等12场标志性战役。推进沿海养殖、工业领域燃煤锅炉“煤改电”。深入实施林水会战，完成造林12万亩，实施重点水利工程61项。严格落实河长制、湖长制，实施重点河流治理工程，确保省控以上断面全面达标。加强水源地保护，确保水质安全。开展土壤污染治理与修复，做好关停矿坑植被保护与恢复治理工作。严守海洋生态红线，推进《海岸带保护与利用管理条例》立法。开展“五大碧海行动”，探索推行湾长制，抓好30公里黄金岸线整治提升工程。严格执行环评审批负面清单制度，推进环保督察整改常态化。

  （四）立足促进城乡融合发展，在推动基本公共服务均等化上加力增效。坚持把城市和乡村摆在同等重要的位置，作为一个整体统筹谋划，努力迈出相互融合、共同发展的新步伐。

  大力实施乡村振兴战略。深化“五大新型载体”建设，推进乡村振兴“十百千”创建工程，建设省级示范乡镇5个、示范村50个，为打造乡村振兴齐鲁样板贡献“日照力量”。新认定市级田园综合体5个、精品民宿5个，推动五莲山—白鹭湾田园综合体争创国家级。加快茶香小镇、航空小镇、艺游小镇、云塑小镇等国家级和省级特色小镇建设，培育创建特色小镇10个。建设乡村记忆馆10个、“非遗工坊”30处、美丽乡村300个。开展高标准农田建设。推进“百县千乡万村”试点示范工程，打造一批“新六产”示范园、示范家庭农场，力争岚山农高区创建为省级，莒县农科园创建为国家级。壮大茶叶、桑蚕、蜂业、中药材、蓝莓等优势产业，加强农产品质量安全监管，提高标准化、规模化、信息化水平，推动向全链条、多业态发展。筹办首届中国（日照）茶博会暨日照绿茶文化节等系列展会。大力实施“头雁工程”，培养政治素质好、能带富、善治理的农村基层组织带头人。引导能人回乡、企业兴乡，共建振兴大业。

  统筹城乡基础设施建设。加快鲁南高铁等重大项目建设，推进厦门路北延、204国道大中修、220省道西延等道路重点工程，构建现代化综合交通体系。开工青峰岭、仕阳水库和日照水库联通工程，实施傅疃河河套、河口地下水库及拦河闸除险加固工程，城西水厂年内建成供水，加快完善现代水网。推动交通、公交、环卫、供气、供水、供电等基础设施向农村延伸。新建改造“幸福公路”1000公里。推进农村饮水巩固提升行动。建设220千伏晨阳、左岭等输变电工程，完成423个农村电网升级改造。稳妥推进合村并居和农房改造。扎实推进农村人居环境整治三年行动，深入实施垃圾治理、“厕所革命”、污水治理等“十大工程”，年内75%的村庄基本完成整治任务，让广大农村美起来。

  提升城市规划建设管理水平。按照“规划有高度、建设有厚度、管理有力度、城市有温度”的要求，推动城市品质化建设、精细化管理。完成城市有轨电车线网等6个专项规划编制，开展日照（石臼湾）中央活力区重点地区城市设计、城市快速路系统详规等规划修编。实施道路优化畅通工程，优先发展公共交通，推进智慧交通管理改造，新建改造城市道路90条。启动城区河道水环境治理三年行动。新建污泥生物处理厂1处，对5座污水处理厂实施升级扩建。扎实推进城中村拆迁改造和老旧小区整治改造，开工棚改安置房5290套。积极发展装配式建筑、绿色建筑。抓好中央活力区等重点片区景观亮化，科学配置学校周边、商业区等重点路段照明。完善城市管理综合考核办法，促进城市管理精细化、规范化、长效化。扎实开展国家生态园林城市创建，大力实施拆迁建绿、拆墙透绿、破硬造绿、见缝插绿工程，建设山海路等城市绿道25公里，打造市民公园、高铁公园等一批城市绿化亮点，让我们的城市处处见风景。

  （五）立足提升群众获得感幸福感安全感，在保障和改善民生上加力增效。增进民生福祉是发展的根本目的，宁可政府开支紧一点，也要保障民生支出、扩大民生投入，织密扎牢覆盖“人的一生”民生保障网，努力让全市人民的生活年年都有新变化。

  促进更加充分就业。把稳就业摆在突出位置，落实稳岗补贴、技能提升补贴、降低企业社会保险费等政策，打好抓稳岗、谋发展、强培训、促双创、兜底线的组合拳。实施高校毕业生就业创业促进计划，做好零就业家庭、农民工、退役军人等重点群体就业，新增城镇就业2.88万人。抓好农民工工资支付保障等工作，依法维护劳动者合法权益。

  优先发展公平而有质量的教育。新建、改扩建普惠性幼儿园76所，加强幼儿园安全规范管理，守护好孩子们的美好童年。新建、改扩建中小学21所，推进义务教育优质均衡发展。高中段入学率达到98%以上。提升特殊教育水平。支持和规范社会力量兴办教育。深入实施乡村教师支持计划。加强师德师风建设，大力弘扬尊师重教社会风尚。

  健全多层次社会保障体系。居民医保补助标准提高到每人每年520元。城乡低保标准分别提高到每人每月590元、470元。城乡特困人员救助供养标准分别提高到每人每月不少于1130元、960元。开展居家和社区养老服务改革，养老院医养结合率达到80%以上，新增社区照料服务设施30处。健全农村留守儿童和妇女、老年人、残疾人关爱服务体系。扩大民生综合保险试点覆盖面。

  推进健康日照建设。积极创建全国健康城市示范市。深化医药卫生体制改革，加快建立现代医院管理制度。发展紧密型医共体和分级诊疗服务模式。推进市医院综合体等重点项目建设。积极引进高水平医疗资源。抓好区县体育设施“五个一”工程，推进乡镇体育公园、行政村户外体育健身广场建设，推动公办学校体育场地设施向社会开放，实现全民健身设施网络和城市社区10分钟健身圈全覆盖。

  繁荣发展文化事业。践行社会主义核心价值观，广泛开展群众性精神文明创建活动。全面推进新时代文明实践中心、县级融媒体中心建设。加强基层综合性文化服务中心建设，推动公共文化服务场所免费开放。做好“中国音乐小金钟”展演、海洋文化节等赛事活动筹办。抓好文艺精品创作。提升黑陶、农民画、沂蒙画派等文化品牌。建成投用市博物馆、美术馆新馆。

  打造共建共治共享社会治理格局。推进依法治市，落实“七五”普法规划。健全完善党政一体法律顾问制度，加快社区矫正基地建设。强化党建引领，加强城乡社区建设，提升基层治理和服务能力。推进智慧社区警务建设，完善社会治安防控体系。持续开展扫黑除恶专项斗争。健全矛盾排查预警和调处化解机制。提升信访工作法治化水平。做好仲裁工作。依法开展第四次全国经济普查。支持国防和军队改革建设，争创全国双拥模范城“六连冠”，推动军民融合深度发展。支持监察委、法院、检察院依法履职。支持群团组织发挥更大作用。做好援疆、扶贫协作等工作。

  四、努力建设人民满意的服务型政府

  打铁必须自身硬。我们将始终坚持以人民满意为最高标准，坚持干字当头、实字托底、好字优先，用担当诠释忠诚，用实干彰显信仰，努力向全市人民交出合格答卷。

  坚决做到“两个维护”。把加强党的政治建设摆在首位，强化“四个意识”，坚定“四个自信”，坚决做到“两个维护”。自觉用习近平新时代中国特色社会主义思想武装头脑、指导实践、推动工作，在思想上政治上行动上同以习近平同志为核心的党中央保持高度一致。坚决贯彻中央决策部署，落实省委、省政府和市委的部署安排，做到令行禁止、使命必达。切实加强党的全面领导，落实好意识形态工作责任制，把党的建设与中心工作拧成一股绳，做到“两手抓、两促进”。

  持之以恒改进作风。把雷厉风行、真抓实干作为政府鲜明的工作风格，言必信、行必果，确保政令畅通、任务落实。向深处、向细处、向实处推进“工作落实年”，建立健全重点工作清单制度、督查机制，量化细化实化政府工作。所有需要政府落实的重点任务、重点事项和民生实事，都建立台账、定期督查、跟踪问效、公开通报。把调查研究作为谋事之基，做好创造性贯彻落实文章，提高抓落实质效。

  始终与人民心连心。牢固树立以人民为中心的发展思想，把增进民生福祉作为一切工作的出发点、落脚点，勤与百姓交心问计，让“民之所望”成为“施政所向”。用好《问政日照》、12345政务服务热线、行风在线平台，畅通政府与公众互动渠道。时刻把群众安危冷暖放在心上，切实解决群众反映强烈的突出问题，既要多办一些成效好、见效快的大事，又要注重从小事入手，把工作做到老百姓心坎上。

  严格依法行政。深化法治政府建设，把政府活动全部纳入法治轨道。深入学习贯彻宪法，自觉忠于宪法、遵守宪法、维护宪法。自觉接受人大依法监督、政协民主监督，认真落实人大讨论决定重大事项、各级政府重大决策出台前向本级人大报告的规定；积极采纳政协民主协商意见建议，促进工作落实。认真办理人大代表建议和政协提案，大力推行“两上网一公开”，积极为人大代表、政协委员履职创造更好条件，广泛听取民主党派、工商联、无党派人士意见。落实重大决策公众参与、风险评估、合法性审查等制度。深入推进政务公开，让权力在阳光下运行。

  恪守清正廉洁。坚定扛起全面从严治党政治责任，加强廉政建设，打造廉洁政府。坚持把纪律规矩挺在前面，筑牢思想“防线”、严守道德“底线”、不碰纪律“红线”，干干净净为人民服务。严格执行中央八项规定及实施细则精神、省委市委实施办法，坚持勤俭办一切事情，从严控制“三公”经费，大力压减一般性支出。深入整治形式主义、官僚主义，严查群众身边腐败和作风问题，形成靠制度管权管事管人的长效机制。

  各位代表！历史的画卷，总是在砥砺中铺展；精彩的华章，总是在实干中书写。让我们更加紧密地团结在以习近平同志为核心的党中央周围，高举习近平新时代中国特色社会主义思想伟大旗帜，在中共日照市委的坚强领导下，锐意进取、埋头苦干，唱响“创新、落实”主旋律，加快建设美丽富饶、生态宜居、充满活力的现代化海滨城市，以高质量发展的优异成绩向新中国成立70周年、日照市建市30周年献礼！
